# Psychological distress as a mediator between workplace violence and turnover intention with caring for patients with COVID-19

**DOI:** 10.3389/fpsyg.2023.1321957

**Published:** 2024-01-08

**Authors:** Sujin Nam, Janet Yuen Ha Wong, Tingxuan Wang, Bomi An, Daniel Yee Tak Fong

**Affiliations:** ^1^School of Health and Environmental Science, College of Health Science, Korea University, Seoul, Republic of Korea; ^2^School of Nursing, Li Ka Shing Faculty of Medicine, The University of Hong Kong, Pokfulam, Hong Kong SAR, China; ^3^School of Nursing and Health Studies, Hong Kong Metropolitan University, Hong Kong, Hong Kong SAR, China; ^4^College of Nursing, Hannam University, Daejeon, Republic of Korea

**Keywords:** COVID-19, nurse, psychological distress, turnover intention, workplace violence

## Abstract

The Coronavirus Disease 2019 (COVID-19) outbreak exacerbated workplace violence and turnover intention among nurses, particularly affecting greater levels of psychological distress. This study aimed to examine psychological distress as a mediator of the relationship between workplace violence and turnover intention among clinical nurses, and to investigate whether caring for patients with COVID-19 moderates this relationship through the lens of the affective events theory. We conducted an online survey of 325 Korean registered nurses (mean age = 30.75; female = 92.6%) who work in clinical settings between August and October 2021 using the convenience sampling method. Psychological distress was measured using the 21-item Depression Anxiety Stress Scale and workplace violence using one dichotomous item adopted from the Workplace violence questionnaire. We measured turnover intention using the six-item Anticipated Turnover Scale. Caring for patients with COVID-19 was determined using one dichotomous item. The research hypotheses assume that the relationship between workplace violence and turnover intention could be mediated by psychological distress and moderated by caring for patients with COVID-19 among Korean nurses. We performed a moderated mediation analysis with workplace violence as the independent variable, turnover intention as the dependent variable, psychological distress as a potential mediator, and caring for patients with COVID-19 as a potential moderator. The analysis revealed that nurses’ psychological distress among 308 nurses had a statistically significant mediating effect on the relationship between workplace violence and turnover intention. Furthermore, caring for patients with COVID-19 had a significant further moderating effect on this relationship. These findings highlight the need for psychological support services for clinical nurses at institutional and organizational levels amidst the ongoing COVID-19 pandemic. It is hoped that these findings can contribute to the development of tailored interventions for nurses caring for patients with COVID-19 to attenuate their psychological distress in a timely and effective manner.

## 1 Introduction

Workplace violence refers to acts or threats of violence, ranging from verbal abuse to physical assault against an employee in their workplace or while on duty [National Institute for Occupational Safety and Health (NIOSH), 2022]. Over half of nurses (58%) in Southeast Asia and Western Pacific countries have encountered workplace violence. The most common types of workplace violence include verbal abuse (64%), intimidation (30%), bullying (25%), physical violence (23%), physical assault (21%), and sexual harassment (12%) (Varghese et al., 2022). Consequently, workplace violence can have detrimental impacts on nurses’ professional outcomes, turnover intention, and physical and psychological health (Varghese et al., 2022). Notably, workplace violence might contribute to nurses’ long-term psychological distress. A longitudinal study involving healthcare providers who were victims of patient violence reported significant psychological distress from 6 to 12 months after violent incident (Lamothe et al., 2021). Another study reported that long-term accumulated exposure to workplace violence, especially bullying, impaired nurses’ psychological hardiness, possibly reducing their ability to cope with stress (Hamre et al., 2020).

Since the Coronavirus Disease 2019 (COVID-19) outbreak, nurses have been susceptible to COVID-19 infection as the need for nursing care increased rapidly during the pandemic. In total, 2,262 nurses had died from COVID-19 in 59 countries as of 31 December, 2020 [International Council of Nurses (ICN), 2021]. The COVID-19 pandemic has adversely affected nurses’ psychological health by presenting considerable local and global inter- and intrapersonal challenges. One study involving 1,783 Korean healthcare staff members reported nurses’ psychological vulnerability, including anxiety and stress, during the first phase of the COVID-19 pandemic (Ahn et al., 2021). According to a systematic meta-analysis review involving 93 studies with 93,112 nurses, over one-third of nurses suffered from psychological distress, including stress (43%), anxiety (37%), and depression (35%), during the pandemic. This exceeded that of their counterparts studied during previous epidemics (Al Maqbali et al., 2021).

Front-line nurses had an increased risk of psychological distress during the pandemic. A study reported that approximately 25% of front-line nurses suffered from psychological distress and risk factors, including working as an emergency department nurse, concern for family members, discrimination, post-traumatic stress symptoms, and negative coping styles (Nie et al., 2020). Another study found that front-line nurses’ stressors, such as fear of infection, patient death, work overload, insufficient preparation, and insufficient support, were positively associated with psychological distress (Lorente et al., 2021). In particular, front-line nurses were adversely affected psychologically by the pandemic while caring for patients with COVID-19. According to a systematic qualitative review, emotional and psychological stress, including anxiety, depression, and fear, were barriers for front-line nurses when caring for patients with COVID-19 (Joo and Liu, 2021). Furthermore, nurses who cared for patients with COVID-19 experienced increased moral distress, which negatively impacts long-term mental health (Lake et al., 2022).

Apart from nurses’ psychological deterioration, the COVID-19 pandemic might have worsened workplace violence against healthcare providers, including nurses, globally (Devi, 2020). Research suggests that 18.5% of front-line clinicians experienced workplace violence during the COVID-19 outbreak, with verbal violence experienced by 16.1% of providers and physical violence by 6.9% (Yang et al., 2021). Two studies involving 1,063 Chinese healthcare personnel reported a workplace violence prevalence of 20.4% during the pandemic (Wang et al., 2020; Yang et al., 2022). The pandemic might have also affected the prevalence of workplace violence among nurses. Nurses’ exposure rates to physical violence and verbal abuse by patients, visitors, and/or family members were 44.4 and 67.8%, respectively (Byon et al., 2022). Mobbing was reportedly the most common experience among 61.6% of nurses, followed by verbal violence in 57.8%, physical violence in 8.4%, and sexual violence in 0.8% (Özkan Şat et al., 2021). Additionally, the COVID-19 pandemic may have exacerbated workplace violence among nurses, as 27.4% of registered nurses (RNs) reported experiencing more violence during the pandemic and 9.5% of RNs had more difficulty reporting violent events than in the pre-pandemic era (Byon et al., 2022). Furthermore, nurses who cared for patients with COVID-19 experienced more physical violence and verbal abuse than those who did not (Byon et al., 2022).

Adverse psychological outcomes have been reported as one of the mediators in the relationship between workplace violence and turnover intention among nurses in both pre-pandemic and pandemic studies. A pre-pandemic study of 6,771 Korean nurses reported that depressive symptoms played a mediating role in the relationship between workplace violence, including verbal violence and sexual harassment, and turnover intention (Pang et al., 2023). Stress was also identified as a mediator in the relationship between workplace violence by patients and turnover intention among nurses working in public hospitals in Pakistan (Laeeque et al., 2018). During the pandemic, a study involving healthcare workers, such as doctors and nurses, found that the relationship between the workplace violence and turnover intention was mediated through social support and psychological distress, including symptoms of depression, anxiety, and stress (Yang et al., 2022).

Understanding the holistic relationships among workplace violence, psychological distress, and turnover intention and the mechanisms associated with these complex relationships is critical for helping nurses properly manage their psychological distress in the context of infectious disease. However, few studies have examined the mediating effects of psychological distress on the relationship between workplace violence and turnover intention among RNs. A pandemic-era study reported that difficulties in identifying whether the increased turnover intention is due to workplace violence, COVID-19, or both may limit the study of this relationship mechanism (Yang et al., 2022). Hence, it is necessary to explore how the relationship between workplace violence and turnover intention works with a hypothesized mediator of psychological distress, adopting a theoretical framework to empirically examine whether psychological distress explains the effect of workplace violence on turnover intention among RNs during the COVID-19 pandemic. The findings of this study will contribute to the redesigning the nursing workforce and the developing pandemic preparedness initiatives.

## 2 Conceptual framework

The affective events theory developed by Weiss and Cropanzano (1996) addresses the structure, causes, and outcomes of affective events at work. This theory accounts for how features of the work environment and work events elicit employees’ affective reactions and influence their affect-driven behaviors, their work attitudes, and judgment-driven behaviors (Weiss and Cropanzano, 1996). To build conceptual models based on this theory, researchers have primarily utilized pathway segments such as work events and affective reactions and work attitudes, or work environment features, work attitudes, and judgment-driven behaviors (Christensen et al., 2023). A scoping review of nursing settings reported that work-related events included tasks and violence. Nurses’ outcomes included their turnover intention, intention to stay, and other behaviors (e.g., avoidance, caring, accident-proneness, and unethical actions) (Christensen et al., 2023).

**Figure 1:**
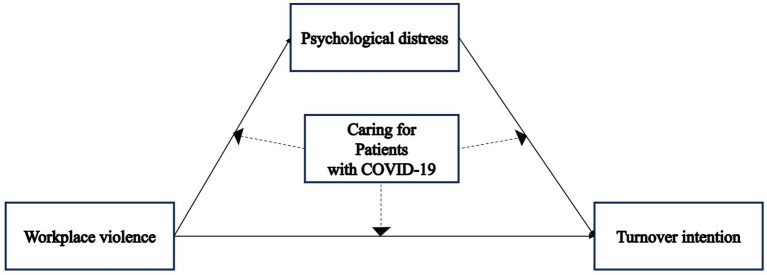
Hypothesized research model.

Based on the literature and the purpose of the present study, the affective events theory (Weiss and Cropanzano, 1996) was adopted as the theoretical basis to support this study’s hypotheses. Thus, a hypothetical model (Figure 1) based on the affective events theory (Weiss and Cropanzano, 1996) was adopted. Specifically, experiencing workplace violence can lead to affective reactions among RNs (i.e., psychological distress) and increase affective-driven behavior or attitudes (i.e., turnover intention). Moreover, nurses caring for patients with COVID-19 have been shown to be more likely to be exposed to workplace violence (Byon et al., 2022) and to have a higher turnover intention levels (Lavoie-Tremblay et al., 2022). Research has also shown that caring for patients with COVID-19 is positively associated with exposure to workplace violence (Xie et al., 2021) and psychological distress (Cho et al., 2021). Therefore, we hypothesized that this relationship may be moderated by caring for patients with COVID-19 as a work environmental feature.

In sum, this study aimed to [National Institute for Occupational Safety and Health (NIOSH), 2022] examine the hypothesized mediating effect of psychological distress on the relationship between workplace violence and turnover intention among nurses who cared for patients with COVID-19 (i.e., a mediation model); and (Varghese et al., 2022) test whether caring for patients with COVID-19 moderates the hypothesized mediation of the relationship between workplace violence and turnover intention in a sample of Korean clinical nurses (i.e., a moderated mediation model). The formulated hypotheses are as follows:

*H1*: Psychological distress would mediate the effect of workplace violence on turnover intention.

*H2*: Caring for patients with COVID-19 would moderate the direct and indirect relationships between workplace violence and turnover intention via psychological distress.

Specifically, caring for patients with COVID-19 would enhance the direct effect of workplace violence on turnover intention (Hypothesis 2a), and enhance the mediating influence of psychological distress on the effect of workplace violence on turnover intention (Hypothesis 2b).

Overall, this study contributes to the pandemic literature by highlighting the role of psychological distress and caring for patients with COVID-19 among clinical nurses. Moreover, focusing on these dimensions is essential for developing strategies and policies to effectively mitigate psychological distress and promote the psychological well-being of nurses in ongoing and future pandemics.

## 3 Methods

### 3.1 Study design

This descriptive cross-sectional study used a convenience sample to explore whether psychological distress mediates the link between workplace violence and turnover intention and whether caring for patients with COVID-19 moderates this relationship. In this study, during the COVID-19 pandemic refers to the period starting with the World Health Organization’s declaration of a global pandemic on 11 March 2020 (World Health Organization, 2020).

### 3.2 Study setting, sample, and data collection

Data were collected from 362 working Korean RNs aged 18 years or older from August through October 2021. Thirty-seven participants were excluded because they did not meet the following criteria: (a) current employment in a clinical setting, (b) directly providing patient care, and (c) clinical nursing experience of more than 6 months. To recruit potential participants, we posted survey flyers that included information regarding the study purpose, research procedures, participation eligibility, research risks and benefits, participation incentives, and a survey link to four major nursing websites: Gandaemo, IMRN, Ganjunmo, and Nurse Forest.

Subjects provided informed consent online after reading the first page of the survey. Eligible participants voluntarily completed the questionnaire using the corresponding institution-licensed Qualtrics survey software.^1^ Only those who agreed to participate in the study could begin the survey. During the data collection period, respondents could withdraw from participation at any time before completing the questionnaire without any consequences. To minimize the potential biases, participants were informed in advance that they could participate in the study regardless of their exposure to workplace violence. Notably, a sample size of 100–400 is recommended in structural equation modeling (Hair et al., 2014). In this study, the “caring for patients with COVID-19 group” included nurses who provided care to those with COVID-19. Others were grouped as the “non-caring for patients with COVID-19 group.”

### 3.3 Outcome measures

#### 3.3.1 Measurements

##### 3.3.1.1 Psychological distress

Nurses’ psychological distress was assessed using the Korean version of the Depression Anxiety Stress Scale (DASS) (Jun et al., 2018). The DASS was developed by Lovibond and Lovibond in 1995 (Lovibond and Lovibond, 1995). The Korean DASS-21, which consists of 21 items, was developed for Korean workers following translation, cross-cultural adaptation, and validation. This scale assesses three domains on a four-point Likert scale with “0” indicating “did not apply to me at all” and “3” indicating “applied to me very much, or most of the time” (Jun et al., 2018). The scores on the three subscales, including depression, anxiety, and stress, are summated for the total score (Jun et al., 2018; Psychology Foundation of Australia, 2023). Higher subscale scores indicate a more severe levels of negative emotional symptoms. A Korean validation study found suitable reliability, content, convergent, and structural validity. Cronbach’s alpha coefficients were 0.93, 0.87, 0.83, and 0.83 for the total score, depression, anxiety, and stress, respectively (Jun et al., 2018). In this study, Cronbach’s alpha coefficients were 0.97, 0.93, 0.90, and 0.90 for the total score, depression, anxiety, and stress, respectively.

##### 3.3.1.2 Workplace violence

The experience of workplace violence was assessed using a dichotomous item from the 10-item validated workplace violence questionnaire (Cheung and Yip, 2017) based on framework guidelines for addressing workplace violence in the health sector (ILO/ICN/WHO/PSI Joint Programme on Workplace Violence in the Health Sector, 2002). This questionnaire reflected the definition of workplace violence suggested by the World Health Organization (Cheung and Yip, 2017). This questionnaire was also used to evaluate workplace violence among full-time nurses working in healthcare settings (Cheung and Yip, 2017). A single item derived from the questionnaire about workplace violence was utilized. The question encompassed workplace violence in the previous 12 months, including verbal abuse, bullying, physical assault, and sexual abuse, and sexual assault (e.g., “Have you encountered any workplace violence in the previous 12 months?”). Participants responded to this question with “yes” or “no.”

##### 3.3.1.3 Turnover intention

Nurses’ turnover intention was measured using the Korean version of the Anticipated Turnover Scale (ATS), a unidimensional scale validated among Korean nurses (Nam et al., 2021). The ATS contains 12 items and was developed by Hinshaw and Atwood (Hinshaw and Atwood, 1980). A meta-analysis of the ATS among RNs in the United States reported an overall mean weighted effect size of 0.89 for reliability and − 0.53 for validity, which demonstrates appropriate reliability and construct validity (Barlow and Zangaro, 2010). The Korean ATS, which includes linguistic and psychometric evaluations, consists of six items, including two reverse-scored components. The assessment employs a seven-point Likert scale, with “1” indicating “strongly disagree” and “7” indicating “strongly agree” (Nam et al., 2021). The total score ranges from 6 to 42. Higher scores denote increased turnover intention. Cronbach’s alpha coefficient and McDonald’s omega coefficient reliability for the Korean validation study were 0.85 and 0.92, respectively (Nam et al., 2021). The Cronbach’s alpha coefficient in this study was 0.84, indicating an acceptable internal consistency.

##### 3.3.1.4 Caring for patients with COVID-19

Caring for patients with COVID-19 was assessed with one question (e.g., “Have you ever provided nursing care to a patient with COVID-19 during the COVID-19 pandemic era?”). The responses were dichotomized into “yes/no” categories.

##### 3.3.1.5 Socio-demographic data, work-related information, and COVID-19-related information

Participants also responded to questions regarding (1) sociodemographic data (i.e., age, sex, marital status, religion, and education level), (2) work-related information [i.e., annual income, nursing experience (years) in the current unit, total years of working experience as an RN, type of working units, type of medical institution, job title, type of work, and employment status], and (3) COVID-19-related information (i.e., work in a COVID-19-related unit, job rotation, changes in regular job duties, overtime work, perceived effectiveness of workplace COVID-19 prevention measures, and COVID-19-associated discrimination during the COVID-19 pandemic era). The COVID-19-related information responses were categorized as “yes/no.”

#### 3.3.2 Statistical analysis

After excluding 17 subjects with missing COVID-19-related information, the descriptive statistics for variables among 308 nurses were estimated. The normality distribution of data variables was assessed using the Shapiro–Wilk test, while the non-normality distribution of data variables was assessed by the Mann–Whitney U test, with a significance level of 0.05, respectively. Spearman’s rank correlation analysis was also performed to identify correlations between the variables. We used the chi-square test and independent t-test to compare the study variables between nurses who delivered care to patients with COVID-19 and those who did not. These variables were then included as covariates in the subsequent moderated mediation analysis. To test the hypotheses about the contingent nature of the mechanism by which workplace violence impacts turnover intention among Korean nurses who cared for patients with COVID-19, a simple mediation model analysis using Model 4 of Hayes’ PROCESS macro version 4.2 (Hayes, 2022) was conducted. This involved 5,000 bootstrap iterations using IBM SPSS Statistics for Window, ver. 27.0 (IBM Corp., Armonk, NY, USA). The moderated mediation analysis was conducted using Model 59 of Hayes’ PROCESS macro (Hayes, 2022) in which the relationship between workplace violence and turnover intention was mediated through psychological distress and further moderated by caring for patients with COVID-19 by controlling previously determined covariates. This involved 5,000 bootstrap iterations to examine whether the direct and indirect paths were moderated by caring for patients with COVID-19. Statistical significance was assessed at an alpha level of 0.05.

### 3.4 Ethical approval

This study was reviewed and approved by the Institutional Review Board of the Ewha Womens University (Reference No. E-202108-0047-02). It met ethical standards including providing comprehensive study information, protecting participants’ rights to withdraw at any time, ensuring confidentiality and anonymity, and obtaining informed consent.

## 4 Results

### 4.1 Nurse characteristics

In total, 308 Korean clinical nurses who were not missing COVID-19-related information were included in this study. The mean age and total duration of professional nursing experience were 30.7 years and 5.7 years, respectively. A total of 286 subjects (92.9%) were female and 221 (71.8%) were unmarried. Additionally, 248 subjects (80.5%) had bachelor’s degrees in nursing and 271 (88.0%) were staff nurses. Approximately 57% worked in medical wards and 13% worked in intensive care units. Nearly 83% reported working in a general hospital or higher. Finally, approximately 84% worked as shift workers and 98% worked as permanent workers (Table 1).

**Table 1:** Sociodemographic characteristics of the participants.

Characteristics		Total (*N* = 308, 100%)	Caring for patients with COVID-9 group (*n* = 129, 41.9%)	Non-caring for patients with COVID-19 group (*n* = 179, 58.1%)	*χ*^2^/*t*	*p*
		*n* (%)/ *M* ± SD	*n* (%)/*M* ± SD	*n* (%)/*M* ± SD		
Age (years)		30.7 ± 5.7	31.3 ± 5.7	30.3 ± 5.6	1.53	0.128
Sex					0.64	0.423
	Female	286 (92.9)	118 (91.5)	168 (93.9)		
	Male	22 (7.1)	11 (8.5)	11 (6.1)		
Marital status					2.73	0.099
	Unmarried	221 (71.8)	99 (76.7)	122 (68.2)		
	Married	87 (28.2)	30 (23.3)	57 (31.8)		
Religion					3.85	0.426
	Protestant	65 (21.1)	23 (17.8)	42 (23.5)		
	Catholic	33 (10.7)	17 (13.2)	16 (8.9)		
	Buddhist	26 (8.4)	13 (10.1)	13 (7.3)		
	None	179 (58.1)	73 (56.6)	106 (59.2)		
	Others	5 (1.6)	3 (2.3)	2 (1.1)		
Education level					3.33	0.343
	Diploma	35 (11.4)	18 (14.0)	17 (9.5)		
	Bachelor	248 (80.5)	100 (77.5)	148 (82.7)		
	Master or above	23 (7.5)	11 (8.5)	12 (6.7)		
	Others	2 (0.6)	0 (0)	2 (1.1)		
Annual income (10,000 KRW)		4358.3 (1103.9) ^b^	4389.0 (1028.9)	4336.1 (1157.6) ^c^	0.41	0.679
Current working unit career (years)		2.7 ± 2.6	2.8 ± 2.4	2.7 ± 2.7	0.22	0.823
Total nursing career (years)		5.3 ± 4.2	5.5 ± 3.9	5.2 ± 4.5	0.66	0.511
Type of working unit					10.40	0.034^a^
	Internal medicine ward	112 (36.4)	43 (33.3)	69 (38.5)		
	Surgical ward	64 (20.8)	24 (18.6)	40 (22.3)		
	Intensive care unit	41 (13.3)	20 (15.5)	21 (11.7)		
	Emergency room	12 (3.9)	10 (7.8)	2 (1.1)		
	Others	79 (25.6)	32 (24.8)	47 (26.3)		
Type of medical institution					14.31	0.003^a^
	Clinic	7 (2.3)	5 (3.9)	2 (1.1)		
	Hospital	47 (15.3)	12 (9.3)	35 (19.6)		
	General hospital	146 (47.4)	74 (57.4)	72 (40.2)		
	Tertiary care hospital	108 (35.1)	38 (29.5)	70 (39.1)		
Job title					0.50	0.779
	Staff nurse	271 (88.0)	112 (86.8)	159 (88.8)		
	Charge nurse	34 (11.0)	16 (12.4)	18 (10.1)		
	Nurse manager	3 (1.0)	1 (0.8)	2 (1.1)		
Type of work					15.59	<0.001^a^
	2-shift work	30 (9.7)	5 (3.9)	25 (14.0)		
	3-shift work	228 (74.0)	110 (85.3)	118 (65.9)		
	Fixed work	50 (16.2)	14 (10.9)	36 (20.1)		
Employment status					2.57	0.109
	Permanent	301 (97.7)	124 (96.1)	177 (98.9)		
	Temporary	7 (2.3)	5 (3.9)	2 (1.1)		
Work in a COVID-19- related unit					158.41	<0.001^a^
	Yes	147 (47.7)	116 (89.9)	31 (17.3)		
	No	161 (52.3)	13 (10.1)	148 (82.7)		
Job rotation					30.04	<0.001^a^
	Yes	104 (33.8)	66 (51.2)	38 (21.2)		
	No	204 (66.2)	63 (48.8)	141 (78.8)		
Changes in regular job duties					31.86	<0.001^a^
	Yes	122 (39.6)	75 (58.1)	47 (26.3)		
	No	186 (60.4)	54 (41.9)	132 (73.7)		
Overtime work					54.65	<0.001^a^
	Yes	175 (56.8)	105 (81.4)	70 (39.1)		
	No	133 (43.2)	24 (18.6)	109 (60.9)		
Perceived effectiveness of workplace COVID-19 prevention measures					3.08	0.079
	Yes	149 (48.4)	70 (54.3)	79 (44.1)		
	No	159 (51.6)	59 (45.7)	100 (55.9)		
COVID-19-associated discrimination					13.23	<0.001^a^
	Yes	103 (33.4)	58 (45.0)	45 (25.1)		
	No	205 (66.6)	71 (55.0)	134 (74.9)		

M, mean; ^a^Significance at the 0.05 level; ^b^*n* = 307 due to one missing data value; ^c^*n* = 178 due to one missing data value.

### 4.2 Descriptive characteristics of the study variables

The results of the Shapiro–Wilk test indicated that turnover intention (*p* > 0.05) was normally distributed while workplace violence (*p* < 0.001) and psychological distress (*p* < 0.001) were not normally distributed. The results of the Mann–Whitney U test showed that there was a significant difference in workplace violence (*U* = 10241.50, *Z* = −2.133, *p* = 0.033) while there was not a significant difference in psychological distress (*U* = 10458.50, *Z* = −1.41, *p* = 0.158) between two groups. The results of Spearman’s rank correlation coefficient showed that workplace violence was positively related to nurses’ turnover intention (*r* = 0.27, *p* < 0.001) and psychological distress (*r* = 0.30, *p* < 0.001). Furthermore, psychological distress was positively related to their turnover intention (*r* = 0.41, *p* < 0.001). Thus, workplace violence, psychological distress, and turnover intention were related. Less than one-third of the 308 nurses reported experiencing workplace violence in the previous 12 months (*n* = 92, 29.9%). A chi-square test showed a statistically significant difference between those caring for patients with COVID-19 and those that were not in association with working unit type (*χ*^2^ = 10.40, *p* = 0.034), medical institution (*χ*^2^ = 14.31, *p* = 0.003), shift work (*χ*^2^ = 15.59, *p* < 0.001), work in a COVID-19-related unit (*χ*^2^ = 158.41, *p* < 0.001), job rotation (*χ*^2^ = 30.04, *p* < 0.001), changes in regular job duties (*χ*^2^ = 31.86, *p* < 0.001), overtime work (*χ*^2^ = 54.65, *p* < 0.001), and COVID-19-associated discrimination (*χ*^2^ = 13.23, *p* < 0.001). To control for their potential effects, the following analyses included the study variables that were statistically significant as covariates.

### 4.3 Testing for a mediation effect

**Figure 2:**
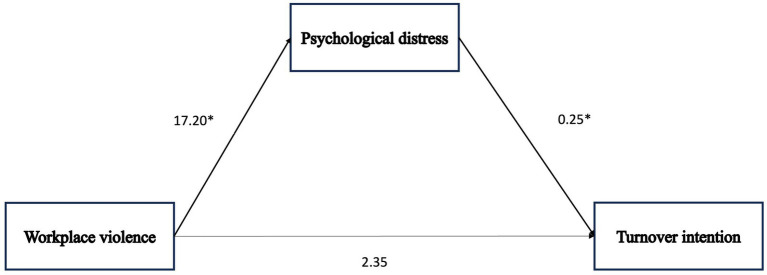
Path diagram illustrating a mediation model with only observed variables in caring for patients with COVID-19 group (*n* = 129). In this model, statistical mediation is examined directly between observed variables. *p < 0.001. *p* values and arrows (→) in bold show statistically significant.

Hypothesis 1 was tested using a mediation model with psychological distress as the mediator. The mediation effect of psychological distress is displayed in Table 2, Model I. Among nurses who cared for patients with COVID-19 (*n* = 129), the coefficient for the indirect effect of the relationship between workplace violence and turnover intention was 4.32 (95% CI: 2.49–6.39). However, the direct effect of workplace violence on turnover intention was not significant (2.35, 95% CI: −0.52–5.21). For the total effect, workplace violence was associated with turnover intention (6.66, 95% CI: 3.88–9.45) (Figure 2; Table 2). Therefore, Hypothesis 1 was supported.

**Table 2:** Direct, indirect, and total effects of variables in the final model.

Model	Path	Direct effect					Indirect effect				Total effect				
		Coefficient	S.E	*t*	LLCI	ULCI	Effect	BSE	95% BCI		Effect	S.E	*t*	LLCI	ULCI
Model I: Caring for patients with COVID-19 group (*n* = 129)	Workplace violence → Psychological distress	17.20^b^	2.61	6.58	(12.03)	(22.37)									
	Workplace violence → Turnover intention	2.35	1.45	1.62	(−0.52)	(5.21)	4.32^a^	0.99	(2.49)	(6.39)	6.66^b^	1.41	4.73	(3.88)	(9.45)
	Psychological distress → Turnover intention	0.25^b^	0.04	5.91	(0.17)	(0.34)									

	Path	Coefficient	S.E	*t*	LLCI	ULCI	Effect	BSE	95% BCI	
Model II: Total sample (*N* = 308)	Workplace violence → Psychological distress	5.08^a^	2.28	2.23	(0.60)	(9.57)				
	Workplace violence → Turnover intention	0.18^b^	0.04	4.50	(0.10)	(0.26)				
	Psychological distress → Turnover intention	1.99	1.14	1.74	(−0.26)	(4.24)				
	Caring for patients with COVID-19 → Psychological distress	−1.81	2.45	−0.74	(−6.64)	(3.02)				
	Caring for patients with COVID-19 → Turnover intention	−2.70	1.59	−1.70	(−5.83)	(0.43)				
Inter 1	Workplace violence × Caring for patients with COVID-19	11.74^b^	3.34	3.52	(5.17)	(18.31)				
Inter 2	Workplace violence × Caring for patients with COVID-19	−0.15	1.80	−0.09	(−3.69)	(3.38)				
Inter 3	Psychological distress × Caring for patients with COVID-19	0.09	0.06	1.52	(−0.03)	(0.20)				
Caring for patients with COVID-19 group (*n* = 129)	Conditional direct effects of the workplace violence on turnover intention	1.83	1.41	1.30	(−0.95)	(4.61)				
Non-caring for patients with COVID-19 group (*n* = 179)		1.99	1.14	1.74	(−0.26)	(4.24)				
Caring for patients with COVID-19 group (*n* = 129)	Conditional indirect effects of the workplace violence on turnover intention						4.44^a^	1.02	(2.62)	(6.60)
Non-caring for patients with COVID-19 group (*n* = 179)							0.91^a^	0.49	(0.06)	(1.95)

BCI, Bootstrapped Confidence Intervals; BSE, Bootstrapped Standard Error; LLCI, Low Limit Confidence Interval; ULCI, Upper Limit Confidence Interval. Model I: A mediation model of psychological distress; Model II: A moderated mediation model of caring for patients with COVID-19. ^a^Significance at the 0.05 level. ^b^Significance at the 0.001 level.

### 4.4 Testing for moderated mediation

**Figure 3:**
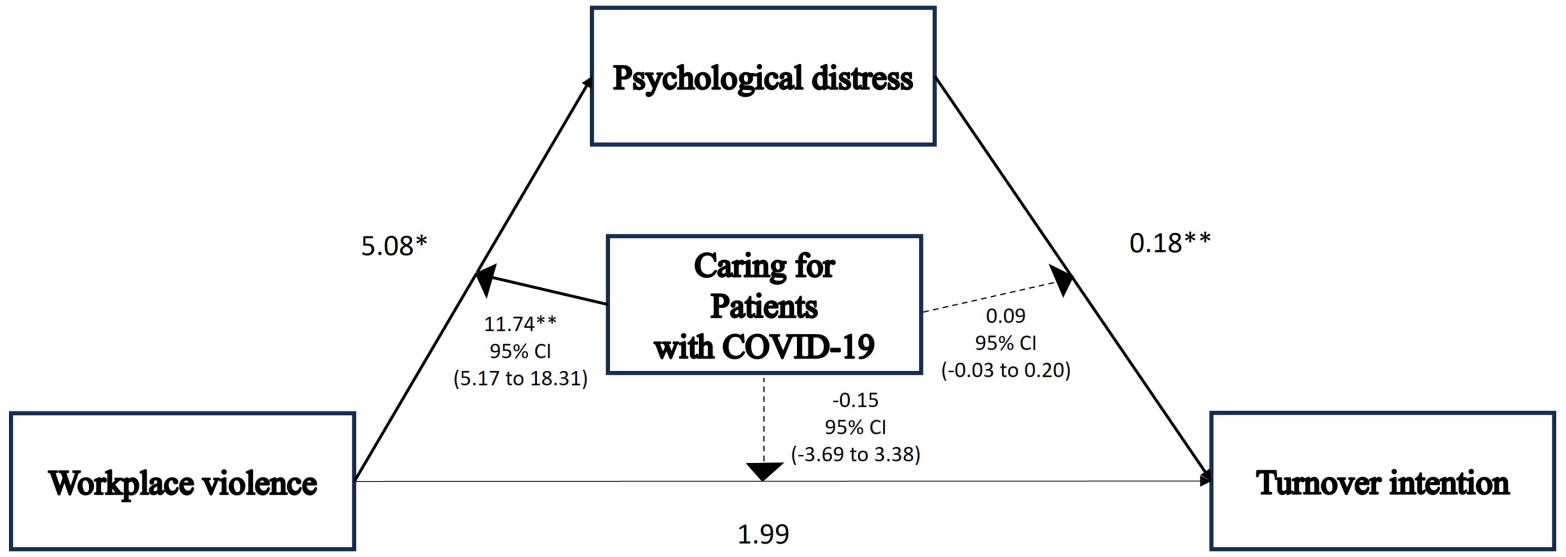
Path diagram illustrating a moderated mediation model with only observed variables in all Korean nurses (*n* = 308). In this model, statistical mediation is examined directly between observed variables. *p < 0.05, **p < 0.001. *p* values and arrows (→) in bold show statistically significant.

Hypothesis 2 was tested using a moderated mediation model that included type of working unit, type of medical institution, type of work, work in a COVID-19-related unit, job rotation, changes in regular job duties, overtime work, and COVID-19-associated discrimination as control variables and caring for patients with COVID-19 as the moderator. Workplace violence was indirectly associated with turnover intention via psychological distress in both nurses who cared for patients with COVID-19 (4.44, 95% CI: 2.62–6.60) and those who did not (0.91, 95% CI: 0.06–1.95) (Model II; Table 2). Workplace violence had a nonsignificant direct effect on turnover intention among nurses who cared for those with COVID-19 (1.83, 95% CI: −0.95–4.61) and those who did not (1.99, 95% CI: −0.26–4.24) (Figure 3; Table 2). This relationship was fully mediated by psychological distress.

The conditional direct effects of workplace violence on turnover intention were nonsignificant among those who cared for patients with COVID-19 (1.83, 95% CI: −0.95–4.61) and those who did not (1.99, 95% CI: −0.26–4.24). Caring for patients with COVID-19 significantly moderated the association between the workplace violence and psychological distress (11.74, 95% CI: 5.17–18.31). The bootstrap CI for the index of moderated mediation was positive (3.54, 95% CI: 1.41–5.85) (Table 2), indicating that the indirect effect of workplace violence on turnover intention through psychological distress differs between nurses who cared for those with COVID-19 and those who did not. Therefore, Hypothesis 2b was supported. In other words, caring for patients with COVID-19 enhanced the mediating effect of psychological distress in the relationship between workplace violence and turnover intention among Korean clinical nurses.

## 5 Discussion

### 5.1 Summary of findings

Using a cross-sectional study, this study examined psychological distress as a mediator of the relationship between workplace violence and turnover intention among clinical nurses. It also tested whether caring for patients with COVID-19 moderates the hypothesized mediation of the relationship between workplace violence and turnover intention.

### 5.2 Mediation model

This study identified the mediating effect of psychological distress on the relationship between workplace violence and turnover intention among clinical nurses. This is consistent with previous literature addressing psychological distress as a mediator, specifically social support and psychological distress (Yang et al., 2022), and occupational stress and burnout (Laeeque et al., 2018). Specifically, this study’s findings are similar to a pre-pandemic study (Laeeque et al., 2018) that examined nurses and stress. This study’s findings are also similar to another pandemic-era study (Yang et al., 2022) that evaluated workplace violence using a dichotomous variable with psychological distress as a mediator.

According to a pre-pandemic study (Laeeque et al., 2018), the mediation effect of stress may be due to nurses’ tendency to consider leaving their organization or profession to avoid occupational stress, thereby increasing their intention to leave (Laeeque et al., 2018). In a pandemic-era study (Yang et al., 2022), according to the conservation of resources theory (Hobfoll, 1989), workplace violence might contribute to a depletion of resources during the COVID-19 pandemic, resulting in increased burnout and stress. Thus, increased psychological distress due to reduced available resources might increase turnover intention to avoid exposure to workplace violence and resource loss (Yang et al., 2022).

Per the findings of a pandemic-era study (Yang et al., 2022), the present findings may be explained by the affective events theory (Weiss and Cropanzano, 1996) and the conservation of resources theory (Hobfoll, 1989). In this study, workplace violence may have exacerbated psychological distress as a negative reaction, and subsequently increased turnover intention as an affective-driven behavior or attitudes among Korean nurses according to the affective events theory. Based on the conservation of resources theory, Korean nurses’ resources might have become scarce due to the exposure to violent incidents in conjunction with infection-related job stressors, which ultimately exacerbated psychological distress. Notably, participants caring for patients with COVID-19 appeared to have suffered from infection-related job stressors including work in COVID-19-related units (89.9%), job rotations (51.2%), changes in regular job duties (58.1%), and overtime work (81.4%). Nurses’ turnover intention might have increased to avoid exposure to workplace violence and reduced resources during the infectious disease crisis. This study developed integrated models to examine the mediating effects of psychological distress in a sample of Korean nurses during the COVID-19 pandemic. These results demonstrate the necessity of devising and implementing strategies and support for clinical nurses related to workplace violence during an infectious disease crisis.

### 5.3 Moderated mediation model

In this study, caring for patients with COVID-19 moderated the indirect effect of workplace violence on turnover intention. To our knowledge, this is the first study to present the moderating role of caring for patients with COVID-19 on the indirect effect of workplace violence on turnover intention. Together, these findings highlight that caring for patients with COVID-19 meaningfully affects the relationship between workplace violence and turnover intention.

Workplace violence was found to significantly and indirectly affected turnover intention among nurses who cared for patients with COVID-19. One possible explanation is that exacerbated moral distress among nurses caring for those with COVID-19 significantly increased psychological distress. During the pandemic, nurses had to balance providing care and maintaining their own health (Rosa et al., 2020). A previous study reported that nurses’ moral distress while providing direct nursing care to patients was positively associated with prolonged psychological distress. This suggests that moral distress could be psychologically traumatic, and that nurses derived anticipated experiencing additional moral distress during the next wave of COVID-19 (Lake et al., 2022).

Among front-line clinicians, moral distress was positively associated with adverse mental health problems including anxiety, depression, post-traumatic stress disorder, and burnout (Smallwood et al., 2021). Based on data from South Korea’s fourth wave of COVID-19 which were used in the present study, the potential positive association between moral distress associated with caring for patients with COVID-19 and psychological distress from workplace violence might contribute to this significant indirect increase in turnover intention among front-line nurses. Governments, organizations, and institutions must actively engage in alleviating nurses’ psychological distress during and following epidemics.

Another explanation for the increased turnover intention linked to workplace violence is the association between the fear of contracting COVID-19 from patients (Hu et al., 2020) and the fear of future workplace violence targeting nurses (Fu et al., 2021). These combined fears could significantly exacerbate front-line nurses’ psychological distress. A Korean study reported that front-line nurses who cared for patients with COVID-19 suffer from psychological distress including anxiety, depression, and fear (Cho et al., 2021). Notably, there were a total of 415 nurses (73.5%) among the 565 healthcare workers who were infected while caring for those with COVID-19 in South Korea (Chung, 2021). In this study, Korean front-line nurses might consider their workplaces’ infection measures to be ineffective (45.7%) and might fear COVID-19 infection. Fear of infection while caring for those with COVID-19, presents a nursing challenge (e.g., becoming infected, dying, and infecting others) (Hu et al., 2020) that could aggravate psychological distress. Accordingly, the increased psychological distress among front-line nurses resulting from the combined fears of COVID-19 and workplace violence might contribute to the significant indirect effect on turnover intention. Further work investigation of the impact of these fears on psychological distress is warranted to improve nurses’ psychological well-being.

This study found a significant interaction effect of caring for patients with COVID-19 in moderating the association between workplace violence and psychological distress. One possible explanation is that Korean nurses might be afraid of becoming infected by violent patients. Notably, a prior study reported more physical violence and verbal abuse experienced by nurses caring for those with COVID-19 than among nurses who did not treat these patients (Byon et al., 2022). Thus, providing care to patients with COVID-19 affects the fear of infection among Korean nurses (Cho et al., 2021), and the increased fear of infection while providing direct care to violent patients may contribute to this significant interaction.

This finding might also be due to the stigma associated with nurses caring for violent patients with COVID-19. A systematic review and meta-analysis investigated the stigmatization of work-related COVID-19 exposure, predominantly self-stigma among healthcare providers (Schubert et al., 2021). Apart from self-stigma, a significant difference in COVID-19-associated discrimination was found between nurses who cared for patients with COVID-19 and those who did not. Increased stigmatization experienced while caring for violent patients may have contributed to this significant interaction effect.

### 5.4 Limitations

This study has some limitations. The use of a cross-sectional online survey does not allow for the determination of causal relationships between workplace violence and turnover intention. However, this study demonstrated the association between workplace violence and turnover intention during the pandemic. Second, incidents could have been under or over-reported due to self-reported data and depended on the nurses’ ability to recall these incidents. This may have been impacted by recall bias. Nevertheless, these biases were minimized using sensitive online surveys. Third, in this study, the assessment considered only 12 months of data during the COVID-19 pandemic and was based on the literature using a one-year prevalence of workplace violence (Liu et al., 2019; Varghese et al., 2022). Considering the data collection period utilized in this study, we assessed different types of workplace violence were assessed over the prior 12 months of the pandemic rather than investigating violence at work during less than 12 months of the pandemic (Yang et al., 2021; Byon et al., 2022). Further longitudinal studies might include information about the accumulation of exposure to workplace violence and changes in affected nursing outcomes trajectories. Fourth, an online survey was conducted to confidentially gather sensitive information. Nurses who participated in the current study were relatively young (mean age: 30.7 years) and had relatively limited work experience (mean: 5.3 years); thus, these results may not be generalizable to all nurse demographic groups. Accordingly, the younger participants sample may be related to selection bias associated with the use of an online survey. Workplace violence was also assessed using a dichotomous, simple question included in the workplace violence questionnaire. Since there are few globally validated scales, developing and validating robust instruments incorporating various types, frequencies, and severities of incidents is necessary to consolidate strong evidence regarding workplace violence. Finally, this study assessed caring for patients with COVID-19 using a dichotomous question without a specific time frame within the context of the pandemic. Thus, future research that considers other aspects, such as the duration of treatment or the number of patients treated by nurses, is warranted.

### 5.5 Implications

This study indicates the need for the development and modification of psychological support services for clinical nurses at the institutional and organizational levels amidst the ongoing pandemic. Workplace violence is a psychological trauma for nurses (Foli, 2022). The development of proactive strategies and interventions to manage psychological distress via professional development, including trauma-informed education, mindfulness-based self-care interventions, and virtual reality-based training, is essential to ensure nurses’ safety and psychological well-being in working environments. These findings empirically demonstrate the mechanisms associated with the relationship between workplace violence and turnover intention via psychological distress between front-line and non-front-line nurses.

Nurses should be encouraged to proactively seek and recive mental health services, including professional psychological counseling [International Council of Nurses (ICN), 2022]. Finally, front-line nurses caring for patients with COVID-19 infection were more vulnerable to workplace violence than non-front-line nurses during the pandemic. Therefore, nurses should be protected through appropriate resources, testing, vaccination, training, and a zero-tolerance policy toward workplace violence [International Council of Nurses (ICN), 2022].

## 6 Conclusion

These results reveal that psychological distress mediates the relationship between workplace violence and turnover intention among clinical nurses in South Korea. Furthermore, a similar mechanism was identified between workplace violence and turnover intention via psychological distress and the difference in psychological distress by workplace violence between front-line and non-front-line nurses. Further research and interventions that consider front-line and non-front-line nurses’ experiences are necessary and require support from policymakers.

## Data availability statement

The datasets presented in this article are not readily available because of university regulations. Requests to access the datasets should be directed to SJN, sujinnam1@connect.hku.hk.

## Ethics statement

The study involving humans was approved by Institutional Review Board of the Ewha Womens University (Reference No. E-202108-0047-02). The study was conducted in accordance with the local legislation and institutional requirements. The ethics committee/institutional review board waived the requirement of written informed consent for participation from the participants or the participants’ legal guardians/next of kin because informed consent does not need to be obtained in written form. All participants provided informed consent online before participating in our study.

## Author contributions

SJN: Conceptualization, Data curation, Formal analysis, Methodology, Original draft, Writing – review & editing. JYW: Conceptualization, Supervision, Writing – review & editing. TW: Methodology, Writing – review & editing. BMA: Data curation, Writing – review & editing. DYF: Supervision, Writing – review & editing.

## Footnotes

^1^ https://www.qualtrics.com/au/
